# Identification of differentially expressed Atlantic salmon miRNAs responding to *salmonid alphavirus* (SAV) infection

**DOI:** 10.1186/s12864-017-3741-3

**Published:** 2017-05-04

**Authors:** Rune Andreassen, Nardos Tesfaye Woldemariam, Ine Østråt Egeland, Oleg Agafonov, Hilde Sindre, Bjørn Høyheim

**Affiliations:** 1 Department of Pharmacy and Biomedical and Laboratory Sciences, Faculty of Health Sciences, Oslo and Akershus University College of Applied Sciences, Pilestredet 50, N-0130 Oslo, Norway; 2 Bioinformatics Core Facility, Department of Core Facilities, Institute of Cancer Research, Radium hospital, part of Oslo University Hospital, Oslo, Norway; 3 Norwegian Veterinary Institute, PB 750 Sentrum, N-106 Oslo, Norway; 4 Department of Basic Sciences and Aquatic Medicine, School of Veterinary Medicine, Norwegian University of Life Sciences, Ullevålsveien 72, 0454 Oslo, Norway

**Keywords:** miRNA, Virus, Atlantic salmon, Innate immune response

## Abstract

**Background:**

MicroRNAs (miRNAs) control multiple biological processes including the innate immune responses by negative post-transcriptional regulation of gene expression. As there were no studies on the role(s) of miRNAs in viral diseases in Atlantic salmon, we aimed to identify miRNAs responding to *salmonid alphavirus* (SAV) infection. Their expression were studied at different time points post infection with SAV isolates associated with different mortalities. Furthermore, the genome sequences of the identified miRNAs were analysed to reveal putative *cis*-regulatory elements, and, finally, their putative target genes were predicted.

**Results:**

Twenty differentially expressed miRNAs (DE miRNAs) were identified. The expression of the majority of these increased post infection with maximum levels reached after the viral load were stabilized or decreasing. On the other hand, some miRNAs (e.g. the miRNA-21 family) showed decreased expression at the early time points post infection. There were significant differences in the temporal expression of individual miRNA associated with different SAV isolates. Target gene prediction in SAV responsive immune network genes showed that seventeen of the DE miRNAs could target 24 genes (e.g. IRF3, IRF7). Applying the Atlantic salmon transcriptome as input 28 more immune network genes were revealed as putative targets (e.g. IRF5, IRF4). The majority of the predicted target genes promote inflammatory response. The upstream sequences of the miRNA genes revealed a high density of *cis*-regulatory sequences known as binding sites for immune network transcription factors (TFs). A high expression in the late phase could therefore be due to increased transcription promoted by immune response activated TFs. Based on the *in silico* target predictions, we discuss their putative roles as early promotors or late inhibitors of inflammation. We propose that the differences in expressions associated with different SAV isolates could contribute to their differences in mortality rates.

**Conclusions:**

This study represents the first steps in exploring miRNAs important in viral-host interaction in Atlantic salmon. We identified several miRNAs responding to SAV infection. Some likely to prohibit harmful inflammation while other may promote an early immune response. Their predicted functions need to be validated and further studied in functional assays to fully understand their roles in immune homeostasis.

**Electronic supplementary material:**

The online version of this article (doi:10.1186/s12864-017-3741-3) contains supplementary material, which is available to authorized users.

## Background

MicroRNAs (miRNAs) are important regulators of gene expression at the post transcriptional level. Pre-miRNAs are transported from the nucleus and processed into two small mature miRNAs (5p and 3p) that usually are 20–24 nt in length. The mature miRNAs are incorporated into the miRNA-induced silencing complex (miRISC). Their function in miRISC are to recognize the target genes by partial base pairing to their transcripts [1, 2]. Due to their role as regulators of gene networks, the majority of miRNAs are highly conserved in vertebrates, but there are also miRNAs that seem to be species specific [3–5].

MiRNAs are involved in regulation of almost all cellular processes including development, growth, maintenance of tissue-specific functions and apoptosis [6–9]. Some miRNAs are also important key regulators of normal immune function and immune responses [10, 11]. Several studies in vertebrates have shown that some miRNAs play important roles in the virus-host interaction following virus infection. Viruses may themselves encode miRNAs (viral miRNAs) that may be involved in cellular reprogramming to promote a cellular environment favorable to viral life cycle. To achieve such an advantage to viral life cycle other viral factors may also repress or induce expression of host miRNAs (cellular miRNAs) [12]. On the other hand, altered expression of cellular miRNAs may also be a consequence of host responses to viral infection where the hosts utilize miRNAs as part of its defense mechanisms [13, 14]. Some of the affected cellular miRNAs have been shown to regulate genes in gene networks associated with innate immune response [15]. The modulation of cellular miRNAs following virus infection could therefore be favorable for either the virus or the host depending on the function of the particular miRNA modulated and the direction of the change in expression [12, 13, 16–18]. As demonstrated in some studies, detailed knowledge about miRNAs, their target genes and the disease mechanisms may potentially lead to the discovery of new diagnostic markers [19] and novel therapeutic approaches [20].

The role of miRNAs following RNA virus infection is less well studied in teleosts than in higher vertebrates. However, some recent studies of economically important teleost species have identified miRNAs responding to viral infection (e.g. [21–24]). We are particularly interested in miRNAs that affect disease development following viral infection in the economically important aquaculture species Atlantic salmon (*Salmo salar L.*). Recently, mature miRNAs and their corresponding miRNA genes were characterized in Atlantic salmon [25, 26], and RT-qPCR methods to study the expression of single miRNAs have been validated [27]. So far, no studies of the putative role(s) of miRNAs in viral disease have been carried out in Atlantic salmon, but one study in rainbow trout (*Onchorhyncus mykiss*) identified miRNAs associated with Viral hemorrhagic septicaemia virus infection [28]. A better understanding of the particular miRNAs showing a change in expression levels, the direction of change (reduced or increased expression) and prediction of their target genes could provide knowledge on the particular role the modulated miRNAs have and which gene regulatory networks are affected.

*Salmonid alphavirus* (SAV), a member of the genus *Alphavirus*, family *Togaviridae*, is a spherical, enveloped, single-stranded positive-sense RNA virus of approximately 60–70 nm in diameter with a 12 kb genome. Pancreas disease (PD) in Atlantic salmon and sleeping disease (SD) in Rainbow trout are caused by infection with SAV [29]. Six SAV subtypes (1–6) have been identified based on the nucleic acid sequences encoding proteins E2 and nsP3 [30]. Two of these, SAV2 and SAV3 have been reported in Norway [31]. Outbreaks of PD leads to increased mortality, reduced growth, affect feed conversion and eventually lead to low product quality. Histological examination usually reveals complete loss of exocrine pancreatic tissue, cardiac myocytic necrosis and inflammation, and degeneration and/or inflammation of skeletal muscle. PD outbreaks leads to large economic losses and is a major concern in the Atlantic salmon aquaculture. A recent challenge study using Norwegian SAV isolates has demonstrated that fish infected with SAV3 have higher mortality than those infected with SAV2. In addition, significant differences in mortality within the SAV3 genotype was observed. The cause for such differences in mortality is unknown [29, 32].

The aim of this study was to identify cellular miRNAs that are differentially expressed (DE miRNAs) following infection with SAV. The expression of DE miRNAs was studied in samples from fish infected by one SAV2 isolate (SAV2-i1) as well as samples from fish infected with two isolates of SAV3 with diverging mortality (SAV3-i4 and SAV3-i6) [32]. Expression of DE miRNAs was measured in each SAV group at three time points post virus challenge. This allowed us to identify miRNAs responding to SAV infection, to study their expression following viral infection by each of the three SAV isolates, and explore whether there were any associations between viral load and miRNA expression. The upstream genome sequences of the differentially expressed miRNA genes were studied in order to identify conserved *cis*-regulatory sequences associated with genes responding to innate immune responses, and, finally, the sequences of the mature DE miRNAs were used to predict their putative target genes. These *in silico* predictions could further contribute to reveal the particular role of the DE miRNAs in the host-virus interaction.

## Results

### RT-qPCR analyses of SAV in cardiac tissue materials

**Fig. 1:**
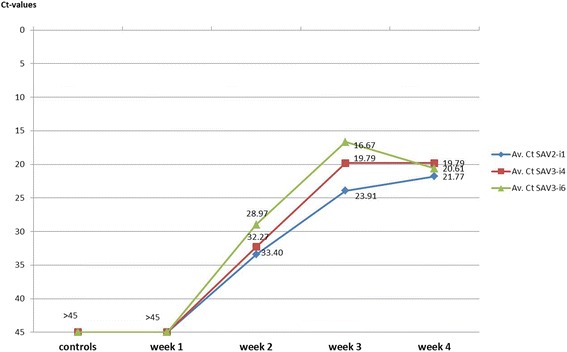
Shows the increase in viral loads assessed by RT-qPCR. The mean Ct-values are given for each of the three groups challenged with SAV2-i1 (*blue*), SAV3-i4 (*dark orange*) or SAV3-i6 (*green*) at week 1–4 poc. The controls are the SAV negative healthy fish sampled at the initiation of the challenge trial (Ct values > 45). At week 1 poc all samples were SAV negative. At week 2 poc the plot show results from only those samples that were SAV positive. All fish sampled at week 3 and 4 poc were SAV positive

The 98 cardiac tissue samples from controls and fish challenged with SAV2-i1, SAV3-i4 and SAV3-i6 collected at week 1–4 post onset of challenge experiment (poc) were successfully analysed for SAV by RT-qPCR. All eight control samples and the nine samples collected at week 1 poc were SAV negative (Ct > 45). Among the samples collected at week 2 poc, five of the nine samples from fish challenged with SAV2-i1 were SAV positive (55%), eight of the nine fish challenged with SAV3-i4 was SAV positive (89%) while all nine fish challenged with SAV3-i6 were SAV positive. All other samples collected at week 3 and 4 poc were SAV positive. Figure 1 illustrates the changes in viral load (assessed by measurements of Ct) in controls and all SAV groups at the four time points (including only the SAV positive samples at week 2 poc).

The results were in agreement with findings in Taksdal et al [32]. In Taksdal’s study all controls were negative (Ct > 45), while at week 2 poc there were 63% SAV positive fish in the SAV2-i1 group, 88% SAV positive fish in the SAV3-i4 group and 99% SAV positive fish in the SAV3-i6 group while all fish from week 3 and 4 poc were SAV positive.

Comparison of Ct values in the SAV positive samples from each of the SAV groups indicated large increases in viral loads from week 2 to week 3 poc. The changes were less pronounced from week 3 to week 4 poc where the obtained Ct values indicated no change in virus load in the SAV3-i4 group, a small increase in the SAV2-i1 group and a small decrease in the SAV3-i6 group. The comparisons of Ct values in the SAV positive samples showed no significant differences at week 2 poc. However, a number of samples were SAV negative at week 2 poc, and most of these were from the SAV2-i1 group. This indicated that the time needed for infection by cohabitation was somewhat longer in the SAV2-i1 group. Ct measurements at week 3 poc did show significant differences (*p* < 0,05) between all three SAV groups (Fig. 1). These Ct-measurements at week 3 poc indicate an approximately ten times higher viral load in the SAV3-i6 group than in the SAV3-i4 group, while there was about seventeen times higher viral load in the SAV3-i4 group than in the SAV2-i1 group. Measurements at week 4 poc revealed smaller differences in Ct values, and only the difference between the SAV2-i1 group and the SAV3-i4 group was significant.

### Identification of DE miRNAs following SAV infection

#### Deep sequencing and identification of DE miRNAs by DESeq2 analysis

Total RNA was successfully extracted from 98 cardiac samples, and six of these were subsequently used for small RNA deep sequencing analysis. These six samples were from two groups; three healthy individuals (control group samples) and three individuals infected with SAV3-i4 (the SAV isolate associated with highest mortality rate in Taksdal et al [32]) collected at week 4 poc (see Materials and Methods). The samples were successfully subjected to next generation sequencing using Illumina Genome Analyzer IIx sequencing platform as described in Materials and Methods. Quality control by FASTQC showed good sequence quality and similar size distributions in all samples. The results from deep sequencing including number of size filtered and adapter trimmed reads, and reads mapped as ssa-miRNAs in each sample are summarized in Additional file 1. Two out of the six samples had a number of reads recovered as miRNAs less than one million. We can not rule out that this may have affected the sensitivity to detect smaller fold changes in low expressed miRNAs. All results have been submitted to the NCBI SRA data base (Genbank SRP048613). Genbank accession numbers to the individual samples are SRR3928621 and from SRR3928623 to SRR3928627.

Reads were successfully mapped as *Salmo salar* miRNAs (ssa-miRNAs) by use of Novoalign (http://www.novocraft.com/products/novoalign/) that aligned reads to a reference miRNAome consisting of the mature 5p and 3p sequences of all known Atlantic salmon miRNAs [25, 26]. The mapped miRNAs along with their read counts were used as input in DESeq2 to reveal differences in relative expression of miRNAs by comparing samples from the group of healthy controls with the SAV3-i4 infected group (see Materials and Methods and [33]). This revealed 20 mature miRNAs that were differentially expressed in the SAV3-i4 group. Eighteen of the miRNAs showed increased expression, while two showed decreased expression. Table 1 shows all miRNAs differentially expressed and the magnitude of the change. Adjusted p-values (Benjamini-Hochberg procedure) with 0.1 as cut-off for significance are given along with the identity of the mature DE miRNAs. The eighteen DE miRNAs that revealed increased expression were ssa-miR-462a-5p and 3p, ssa-miR-462b-5p, ssa-miR-731-5p and 3p, ssa-miR-146b-5p, ssa-miR-146a-3p, ssa-miR-146a-3-3p, ssa-miR-21a-1-3p, ssa-miR-21a-2-3p, ssa-miR-21b-3p, ssa-miR-181c-5p, ssa-miR-223-5p, ssa-miR-1338-3p, ssa-miR-155-5p, ssa-miR-92a-3-5p, ssa-miR-7132-5p and 3p. The expressions of these miRNAs were from approximately 3x to 19x higher in the SAV3-i4 infected group. Two miRNAs, ssa-miR-734-3p and ssa-miR-2188-3p, revealed decreased expression which was approximately 4x (ssa-miR-734-3p) and 2x (ssa-miR-2188-3p) lower in the SAV3-i4 infected group.

**Table 1:** DE miRNAs revealed by DESeq2 and RT-qPCR analysis in SAV3-i4 infected individuals at week 4 poc

Mature miRNA	SAV expression^1^	log2 fold change^2^	DESeq2 P-adj.^3^	log2 f.c. RT-qPCR^4^	RT-qPCR P-adj.^5^	Family^6^
ssa-miR-462a-5p	increase 13x	3.71	1.93x10^-5^	2.58	3.0x10^-3^	462
ssa-miR-462a-3p	increase 19x	4.30	2.41x10^-5^	3.12	3.0x10^-3^	462
ssa-miR-462b-5p	increase 3x	1.75	0.07	−	−	462
ssa-miR-731-5p	increase 12x	3.58	1.79x10^-3^	2.63	3.0x10^-3^	731
ssa-miR-731-3p	increase 8x	3.04	1.64x10^-3^	2.28	3.0x10^-3^	731
ssa-miR-146b-5p	increase 14x	3.82	1.17x10^-4^	2.38	3.0x10^-3^	146
ssa-miR-146a-3p	increase 6x	2.64	0.03	2.99	3.0x10^-3^	146
ssa-miR-146a-3-3p	increase 8x	3.00	0.03	−	−	146
ssa-miR-21a-1-3p	increase 10x	3.27	4.3x10^-3^	3.31	3.0x10^-3^	21
ssa-miR-21a-2-3p	increase 8x	3.16	0.02	2.21	3.0x10^-3^	21
ssa-miR-21b-3p	increase 5x	2.36	0.06	2.33	3.0x10^-3^	21
ssa-miR-181c-5p	increase 4x	2.14	0.09	1.42	0.011	181
ssa-miR-223-5p	increase 5x	2.36	0.06	2.46	3.0x10^-3^	223
ssa-miR-1338-3p	increase 3x	1.65	0.06	1.61	3.0x10^-3^	1338 (1388)
ssa-miR-155-5p	increase 3x	1.72	0.06	0.58	0.1	155
ssa-miR-92a-3-5p3	increase 10x	3.4	0.06	−	−	92
ssa-miR-7132-3p	increase 3x	1.79	0.06	1.88	3.0x10^-3^	7132
ssa-miR-7132-5p	increase 4x	2.11	0.07	1.06	0.02	7132
ssa-miR-734-3p	decrease 4x	-2.09	0.07	-0.19	0.96	734
ssa-miR-2188-3p	decrease 2x	-1.08	0.10	-0.95	3.0x10^-3^	2188

^1^Direction and approximate relative change (times fold change) of mature miRNAs in SAV infected tisssues from DESeq2 measurements

^2^Log2 fold change from DESeq2 analysis of deep sequencing samples

^3^Adjusted *p*-values from DESeq2 analysis. 0.1 was used as threshold for significance

^4^Log2 fold change (∆∆Ct-method) from RT-qPCR analysis

^5^ *P*-values from RT-qPCR after adjustment according to Benjamini-Hochberg procedure. 0.05 was used as threshold for significance

^6^Those with identical numbers belong to same miRNA gene family or are corresponding 5p and 3p from same miRNA gene

The 20 DE miRNAs identified were derived from only 12 miRNA gene families (Table 1). MiRNAs that belong to the same gene family (e.g. ssa-miR-462a and ssa-miR-462b) are believed to be evolutionary very closely related and often co-expressed [34]. Thirteen DE miRNAs were from miRNA genes that are conserved through evolution and present also in higher vertebrates. Seven of the DE miRNAs were from four miRNA genes (miRNA-7132, miRNA-734, miRNA-462 and miRNA-731) that have been discovered in teleosts only (http://www.mirbase.org/). Furthermore, two of these genes, miRNA-731 and miRNA-462a, are located within the same miRNA gene cluster in the Salmon genome [25].

#### Validation of DESeq2 results by RT-qPCR analysis

DESeq2 is a robust and widely used statistical tool for quantitative analysis of deep sequencing data. However, it is still common practice to validate any findings by use of an RT-qPCR method that can target each of the differentially expressed mature miRNAs individually. Such independent validations also allow for measurements of their expression in a larger population sample. Thus, for the purpose of validating our results we developed RT-qPCR assays to measure expression of the different DE miRNAs identified by DESeq2 analysis. Two miRNAs were not included in this validation (ssa-miR-462b-5p and ssa-miR-146a-3-3p) as two other miRNAs (ssa-miR-462a-5p and ssa-miR-146a-3p) with very similar mature sequences from the same miRNA families were included. RT-qPCR assays were successfully developed for 17 DE miRNAs. The miRNA specific primer sequences along with measurements of assay performances (R-squared and efficiency) for all these RT-qPCR assays are given in Additional file 2. One miRNA assay (ssa-miR-92a-3-5p) did not pass the validation of the RT-qPCR assay due to non-specific amplification and was not analysed further.

The comparative Ct method (ΔΔCt-method) [35] were used to measure the relative difference in miRNA levels between the healthy controls (*n* = 8) and SAV3-i4 positive individuals (*n* = 9) at week 4 poc. The results are summarized in Table 1. The log2 fold changes (ΔΔCt) as well as adjusted p-values from each miRNA analysed by RT-qPCR is given. The results from RT-qPCR analysis agreed very well with the results from DESeq2 analysis for 15 of the 17 miRNAs tested. They all showed similar changes in expression which was significantly different compared to the controls (significant threshold *p* = 0.05). The two remaining miRNAs (ssa-miR-155-5p and ssa-miR-734-3p) did reveal differences in the same direction as those from deep sequencing, but they were less pronounced and not significant. Considering the good agreement between DESeq2 analysis and RT-qPCR for the other 15 miRNAs it may be possible that the lack of correlation between the two methods for these two miRNAs was due to suboptimal RT-qPCR assay performance. An efficiency in the lower range for these two RT-qPCR assays (Additional file 2) support that the RT-qPCR was the method providing the less accurate measurements of fold changes for these two miRNAs.

**Fig. 2:**
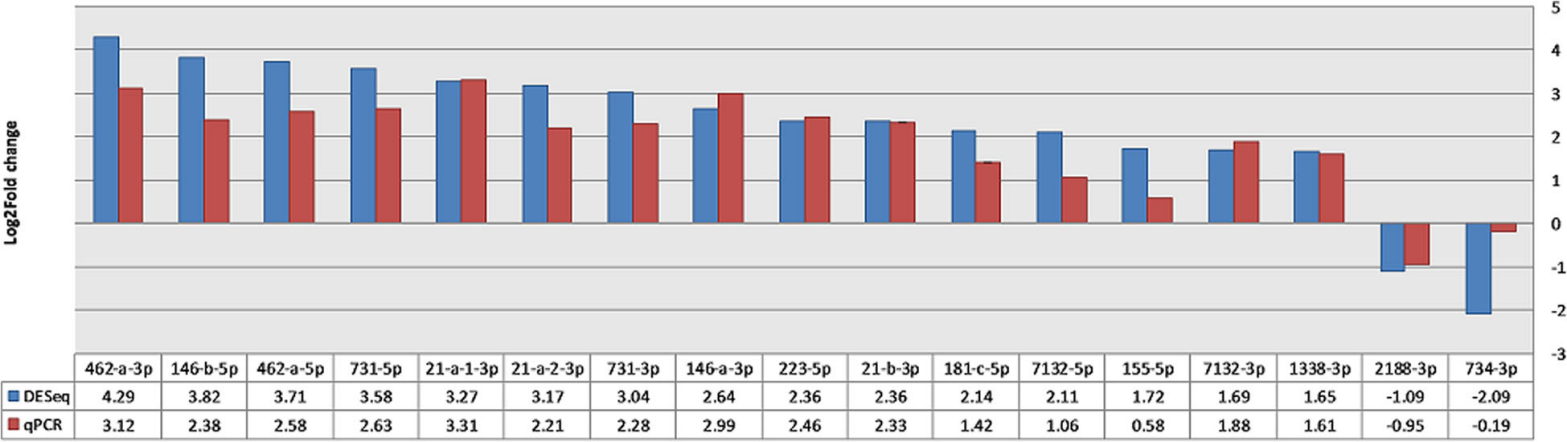
Shows the relative change in the 17 miRNAs initially detected by DESeq2 analysis and subsequently analysed by RT-qPCR. The *blue bars* indicate the log2 fold changes from DESeq2 analysis. The *red bars* indicate the log2 fold changes from RT-qPCR analysis. Results from DESeq2 and RT-qPCR for same miRNA are grouped together. Below the figure is a table with log2 fold changes given for each of the miRNAs from both DESeq2 and RT-qPCR analysis

Figure 2 shows the results (log2 fold changes) for the 17 miRNAs that were analysed by both deep sequencing (DESeq2) and RT-qPCR (ΔΔCt-method). The figure illustrates the good agreement between the relative expression changes detected by the two methods. Spearman’s nonparametric test for correlation showed ρ = 0.83 (*p* = 0.001). The good correlation between the methods also demonstrates that the RT-qPCR assays were suitable for measurements of miRNA expression in all SAV positive samples collected (three SAV groups, different time points).

### RT-qPCR analysis of miRNA expression in SAV2-i1, SAV3-i4 and SAV3-i6 groups

The individuals in the three groups challenged with either SAV2-i1, SAV3-i4 or SAV3-i6 were successfully analysed by RT-qPCR at the three time points (week 2, 3 and 4 poc). The relative changes in expression (ΔΔCt) in the 17 DE miRNAs were estimated by comparison to the control group. A complete overview of all results from RT-qPCR analysis showing the magnitude of change in each SAV-group at each of the time points along with measurements of significance (adjusted *p*-values) is given in Additional file 3.

**Fig. 3:**
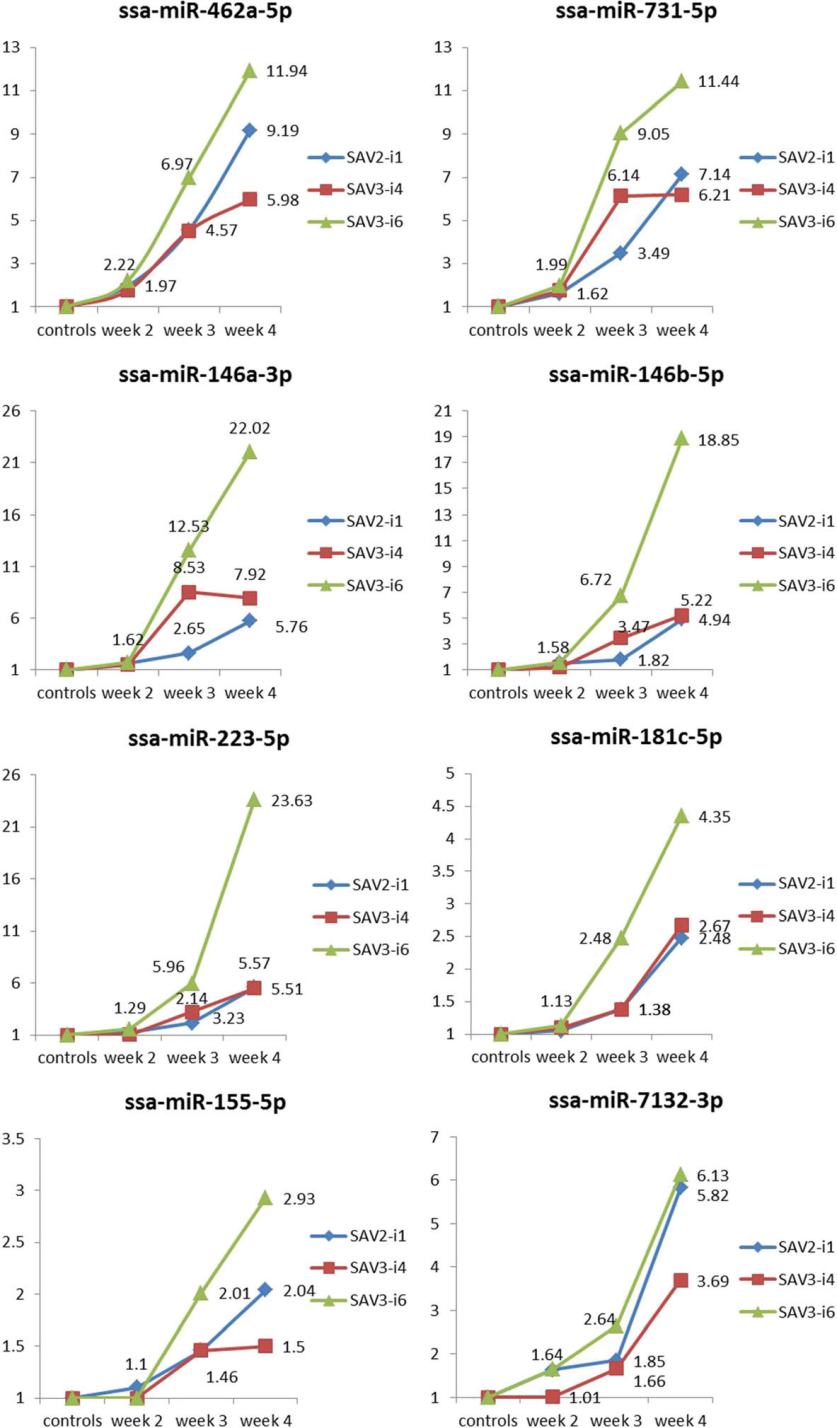
Shows the relative changes in miRNA expression at all time points for each of the SAV groups for the following miRNAs: ssa-miR-462a-5p, ssa-miR-731-5p, ssa-miR-146a-3p, ssa-miR-146b-5p, ssa-miR-223-5p, ssa-miR-181c-5p, ssa-miR-155-5p and ssa-miR-7132-3p. The corresponding mature miRNAs from the miRNA gene cluster miRNA-462/731 (ssa-miR-462a-3p and ssa-miR-731-3p) showed very similar results, and for simplicity reasons these are not given in the figure, but in Additional file 3

The results from all time points showed that there was only one miRNA (ssa-miR-734-3p) that could not be verified by RT-qPCR as significantly different from the controls in any of the SAV groups. A majority of the miRNAs showed increased expression across the time points with the largest increases between week 3 and 4 poc, and the higher level of expression at the latest time point (week 4 poc) (Fig. 3). Viral load, on the other hand, showed the largest increases from week 2 to week 3 poc and modest changes from week 3 to week 4 poc (Fig. 1). Comparing the changes in miRNA expression with the changes in viral load, the expression of most of the miRNAs seem to mirror the change in viral load as if the large increases to the highest expression level at week 4 poc were a time delayed response to the large increase in viral load from week 2 to week 3 poc.

**Fig. 4:**
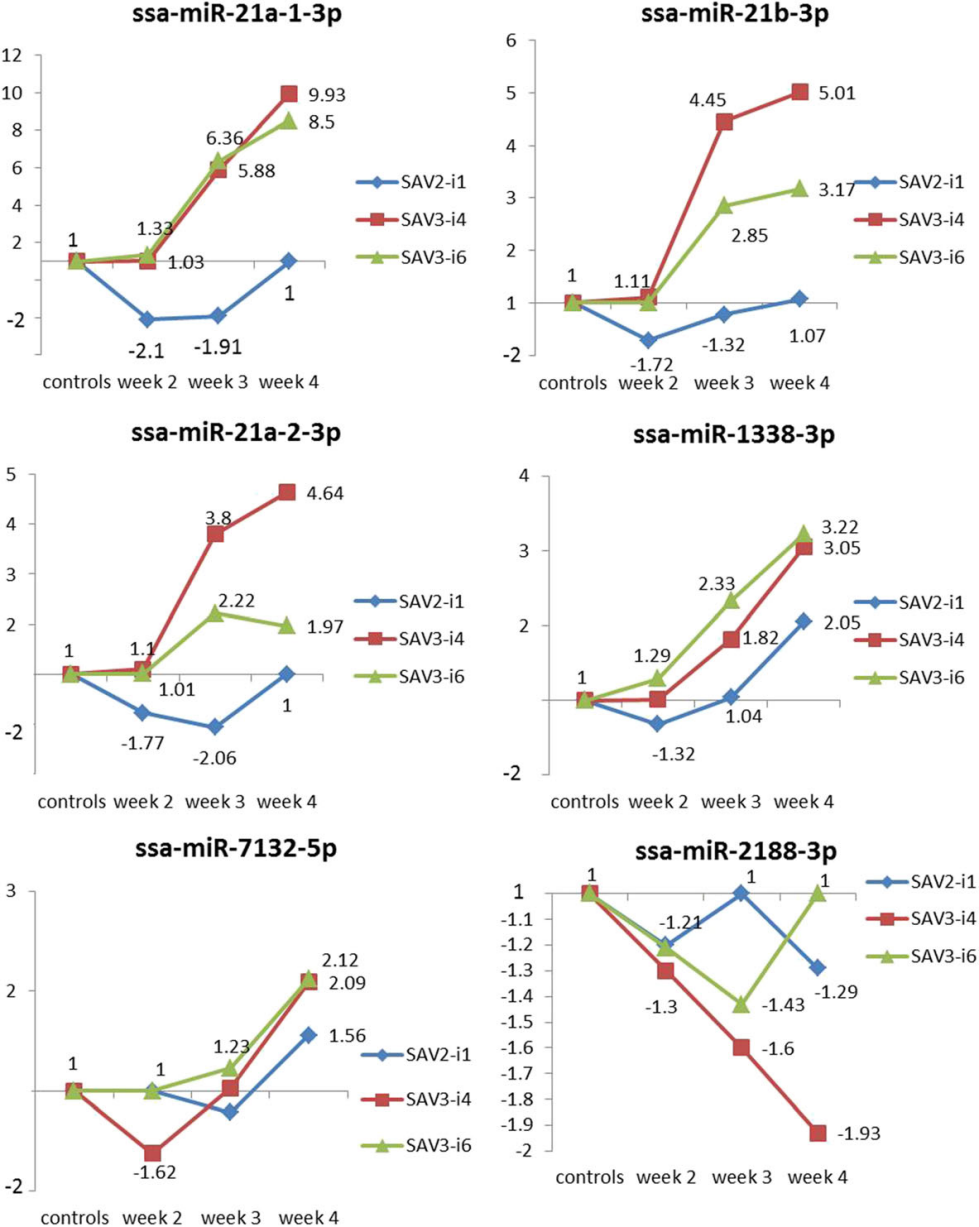
Shows the relative changes in expression for ssa-miR-21a-1-3p, ssa-miR-21b-3p, ssa-miR-21a-2-3p, ssa-miR-1338-3p, ssa-miR-7132-5p and ssa-miR-2188-3p at all time points for each of the SAV groups

There were, however, differences in magnitude of the increases between groups. There were also differences in the direction of change between different miRNAs as some of the miRNAs revealed other changes than increases only (e.g. the miRNA-21 family and ssa-miR-1338-3p) (Fig. 4). These differences between groups are interesting as fish infected with either SAV2-i1, SAV3-i4 or SAV3-i6 have revealed differences in mortality rates [32].

#### miRNAs with higher expression level in the SAV3-i6 group than the SAV3-i4/SAV2-i1 groups

Ten of the 16 DE miRNAs (ssa-miR462a-5p and 3p, ssa-miR-731-5p and 3p, ssa-miR-146a-3p, ssa-miR-146b-5p, ssa-miR-223-5p, ssa-miR-181c-5p, ssa-miR-155-5p and ssa-7132-3p) showed increases across week 2, 3 and 4 poc in all SAV groups. Figure 3 illustrates the increases for eight of these miRNAs. The 5p and 3p mature miRNAs of ssa-miRNA-462a and ssa-miRNA-731-5p genes showed very similar patterns (see Additional file 3) and only the 5p mature miRNAs were included in Fig. 3

The expressions were significantly different from the controls at week 3 and 4 poc in all three SAV groups in seven miRNAs; ssa-miR462a-5p, ssa-miR462a-3p, ssa-miR-731-5p, ssa-miR-731-3p, ssa-miR-146a-3p ssa-miR-223-5p and ssa-7132-3p. The miRNAs ssa-miR-155-5p, ssa-miR-181c-5p and ssa-miR-7132-3p were the miRNAs with the smallest increases in expression, and they were significantly different to controls in all groups at week 4 poc.

Common to all these ten miRNAs was that the SAV3-i6 group revealed a higher expression than the SAV3-i4 and SAV2-i1 groups at week 3 and 4 poc. A comparison showed that in the four miRNAs ssa-miR-731-5p, ssa-miR-146a-3p, ssa-miR-146b-5p and ssa-miR223-5p, there was a significant difference in expression in the SAV3-i6 group *versus* both the SAV2-i1 and SAV3-i4 groups at week 4 poc. Four of the other miRNAs ssa-miR-462a-5p, ssa-miR-731-3p, ssa-miR-155-5p and ssa-7132-3p showed significantly higher expression in the SAV3-i6 group than in the other SAV3 group (SAV3-i4) at week 4 poc. The SAV3-i4 and SAV2-i1 groups showed, in general, a more similar expression to each other than to the SAV3-i6 group (Fig. 3).

#### miRNAs with decreased expression at early time points

The miRNAs from the miRNA 21 family (ssa-miR-21a-1-3p, ssa-miR-21b-3p and ssa-miR-21a-2-3p) and miR-1338-3p revealed a decreased expression across the early time points in the SAV2-i1 group. This was in contrast to the two other groups that showed unchanged or increased expression compared to controls at week 2 and 3 poc (Fig. 4). Interestingly, the SAV3-i4 group with the highest mortality rate also showed a higher expression of the three miRNAs from the miRNA 21 family than the SAV3-i6 group.

The changes in expression in ssa-miR-1338-3p were small in all SAV groups, but only the SAV2-i1 group showed a reduced expression, and the SAV2-i1 group was significantly different from SAV2-i6 at week 3 poc (Fig. 4). Ssa-miR-7132-5p and ssa-miR-2188-3p did both show a decreased expression at one or more time point in the SAV3-i4 group (Fig. 4). Ssa-miR-7132-5p showed a significant decrease at week 2 poc while ssa-miR-2188 revealed a decrease in expression across all time points in the SAV3-i4 group that was significantly different from both the controls and the SAV3-i6 group at week 4. Ssa-miR-734-3p showed a small decrease that was not significantly different to controls in any of the SAV groups (Additional file 3). However, this miRNA may have been more accurately measured in the DESeq2 analysis (4x times decrease, Table 1).

### Prediction of miRNA target genes

The four miRNA target prediction tools RNAhybrid, TargetSpy, PITA and Miranda [36, 37] were applied to predict the target genes. Two different approaches were used for selecting the genes used as input. First, we used a knowledge based approach where immune responsive genes known to respond to SAV infection [38, 39] were used as input. A few immune relevant genes known to be targets of the identified DE miRNAs in other vertebrates [40–45] were also included. Thus, a total of 50 genes (Additional file 4) were analysed in this first approach. Then we performed a second target gene prediction without any prior selection based on function or response to SAV. Applying such a holistic approach all known Atlantic salmon transcripts in the Refseq database of Genbank were considered putative targets and used as input. The latter approach would predict a large number of false positives [46], but could also potentially identify immune response related genes not previously identified as differentially expressed following SAV infection that are true targets.

The results from analyzing genes known to respond to SAV infection showed that 24 of the genes were predicted as putative targets by at least three of the prediction tools applied. The genes along with the mature miRNAs predicted to target each of these genes are shown in Table 2. Thirteen of the predicted target genes revealed multiple target sites to several different DE miRNAs in their 3’UTR’s. Three of these genes, neutrophil cytosolic factor 1, myeloperoxidase and IKKa, were predicted to have target sites to five of the DE miRNAs. One of the miRNAs (miR-223-5p) predicted to target IKKa has also been reported as targeting this gene in other vertebrates [43].

**Table 2:** Results from prediction of target genes among a set of SAV responsive immune network genes

Gene	Gene function/relevance to SAV infection^1^	Predicted by four search tools^2^	Predicted by three search tools^3^	Reference^4^
Neutrophil cytosolic factor 1	inflammatory response, superoxide anion and cell death, increased expression in SAV	miR-92a-3-5p, miR-181c-5p	miR-146a-3-3p, miR-146a-3p, miR-155-5p	Johansen et al., [38]
VHSV inducible like protein (VIG-B319)	virus responsive, increased expression in VHSV and SAV	miR-155-5p, miR-223-5p	miR-146b-5p, miR-1338-3p	Johansen et al., [38]
Jeltraxin	similar to CRP, innate immunity pathway, increased expression in SAV	miR-146b-5p	miR-181c-5p	Johansen et al., [38]
Interferon-inducible protein Gig2-7-like	virus responsive, increased expression in SAV	−	miR-462a-3p	Johansen et al., [38]
Nuclear factor of kappa light polypept gene enhancer in B-cells 2 (NFkB2)	miR-223 target in other vertebrate, innate immune system, inflammatory response	miR-462a-3p	miR-146b-5p	Aziz et al., [43]
Myeloperoxidase	promote inflammation, innate immunity pathway, increased expression in SAV	miR-462a-3p, miR-146b-5p	miR-223-5p, miR-734-3p, miR-2188-3p	Johansen et al., [38]
Macrosialin precursor CD68	virus responsive, increased expression in SAV	miR-462a-3p, miR-731-5p	miR-181c-5p	Johansen et al., [38]
ATP-dependent RNA helicase DHX58 (RIG-I-like 3)	virus responsive, innate immunity pathway, increased expression in SAV	−	miR-731-5p	Johansen et al., [38]
Interferon regulatory factor 7 (IRF7)	transcription factor activating innate immunity pathway, increased expression in SAV	miR-731-5p	−	Xu et al., [39]
Interferon regulatory factor 3 (IRF3)	transcription factor activating innate immunity pathway, increased expression in SAV	miR-21a-2-3p, miR-21b-3p	miR-181c-5p, miR-734-3p	Xu et al., [39]
C–C motif chemokine 19	inflammatory response, innate immunity pathway, increased expression in SAV	−	miR-731-3p, miR-223-5p, miR-1338-3p	Johansen et al., [38]
Interferon-inducible protein Gig2-2-like	virus responsive, increased expression in SAV	miR-181c-5p	−	Johansen et al., [38]
Inhibitor of nuclear factor kappa-B kinase subunit alpha-like (IKKa)	miR-223 target in other vertebrate, innate immune system and inflammatory response	miR-181c-5p (multiple sites)	miR-223-5p, miR-7132-5p, miR-1338-3p, miR-734-3p	Aziz et al., [43]
Cholesterol 25-hydroxylase-like protein A	virus responsive, innate immunity pathway, increased expression in SAV	miR-181c-5p	−	Johansen et al., [38]
Complement factor D (CFAD)	inflammatory response, innate immunity pathway, upregulated in SAV	−	miR-223-5p	Johansen et al., [38]
Interleukin-10 receptor beta chain precursor	Cytokine-mediated signaling pathway, immune response, upregulated in SAV	−	miR-21a-2-3p, miR-146a-3-3p, miR-146a-3p, miR-223-5p	Johansen et al., [38]
SOCS1	antiviral innate immune response	−	miR-223-5p	Taganov et al., [45]
Fish virus induced TRIM-3	Virus responsive, antiviral innate immunity, upregulated in SAV	−	miR-181c-5p	Johansen et al., [38]
Forkhead box protein O1-A-like (FOXO1)	miR-223 target in other vertebrate, immune system signaling pathways, promote cell death	−	miR-2188-3p	Aziz et al., [43]
Complement C1qB subcomponent	inflammatory response, innate immunity pathway, increased expression in SAV	−	miR-21a-1-3p, miR-223-5p, miR-2188-3p (multiple sites)	Johansen et al., [38]
Barrier-to-autointegration factor	known to be exploited by retrovirus, increased expression in SAV	−	miR-21a-2-3p, miR-21b-3p	Johansen et al., [38]
Viperine (RSAD2)	virus responsive, innate immunity antiviral, increased expression in SAV	−	miR-223-5p, miR-734-3p	Johansen et al., [38]
Interferon gamma 1	virus responsive, cytokine	−	miR-734-3p	Sun et al., [40]
Interferon gamma 2	virus responsive, cytokine	−	miR-734-3p	Sun et al., [40]

^1^Key words about gene function are based on gene function description in the http://www.uniprot.org/uniprot/ database. ^2^DE-miRs predicted to target the gene by all the four target search tools used (RNA-hybrid, TargetSpy, PITA, Miranda)

^3^DE-miRs predicted to target the gene by three of the four target search tools used. ^4^Relevance to SAV is based on findings in the references given in this column

On the other hand, 17 of the 20 DE miRNAs (Table 1) could target at least one gene. Additional file 5 illustrate such a duplex between a miRNA and the 3’UTR of the target gene. This particular match is between ssa-miR-21b-3p and the 3’UTR of the Atlantic salmon IRF3 transcript. The distribution of miRNAs that were predicted to target multiple genes showed that 13 of the miRNAs; ssa-miR-462a-3p, ssa-miR-731-5p, ssa-miR-146b-5p, ssa-miR-146a-3p, ssa-miR-146a-3-3p, ssa-miR-181c-5p, ssa-miR-223-5p, ssa-miR-734-3p, ssa-miR-21a-2-3p, ssa-miR-21b-3p, ssa-miR-1338-3p, ssa-miR-155-5p and ssa-miR-2188-3p were predicted to target several genes. The miRNAs predicted to target the largest number of genes were the two miRNAs ssa-miR-223-5p and ssa-miR-181c-5p. These miRNAs showed target matches to nine and eight of the genes, respectively (Table 2). The miRNA showing largest number of target gene matches (miRNA-223) has been suggested as a diagnostic marker for inflammation in vertebrates [43]. Two of the mature miRNAs, ssa-miR-462a-3p and miR-731-5p, that originate from the same miRNA gene cluster [25], were both predicted to target the 3’UTR of the same gene (macrosialin, Table 2). Two miRNAs showed down regulation at the later time points; ssa-miR-2188-3p and ssa-miR-734-3p (Fig. 2). Ssa-miR-2188-3p showed a match to FOXO1 (cell death promotor) while ssa-miR-734-3p showed matches to interferon gamma (virus responsive cytokine), inhibitor of nuclear factor kappa-B kinase (IKKa) and viperine.

The conserved miRNAs miR-146, miR-155 and miR-223 have been predicted to target important immune system response genes in other vertebrates (e.g. IRAK1, TRAF6, SOCS1 and STAT3) [40–43, 45]. The same Atlantic salmon miRNAs (ssa-miR-146, ssa-miR-155 and ssa-miR-223) did not reveal any target site matches to the Atlantic salmon orthologs of these genes (Additional file 4). On the other hand, the teleost specific miRNA, miR-731-5p, has been shown to target IRF7 in Japanese flounder [44]. This gene (IRF7) was also predicted as a target gene in Atlantic salmon where ssa-miR-731-5p showed a target site match to the 3’UTR of the Atlantic salmon IRF7 transcript (Table 2). Likewise, the miR-223-5p has been predicted to target IKKa in humans [43] and did also reveal target site in the 3’UTR of the Atlantic salmon IKKa transcript. The conservation of these target sites among distant vertebrate species indicates that these two genes are true targets of ssa-miR-731-5p and ssa-miR-223-5p. In summary, the findings from target gene prediction in the set of SAV responsive genes indicated that 24 genes, many that are key participants in the immune system networks and known to respond to SAV infection, may be negatively regulated by the DE miRNAs identified in this study.

The target gene predictions applying all Atlantic salmon mRNAs (Refseq, Genbank) as input (holistic approach) generated matches to a total of 1335 transcripts. Each DE miRNA was predicted to target several transcripts with numbers ranging from 30 transcripts (miR-7132-3p) to 389 (miR-181c-5p). The complete results from this analysis, showing the predicted target transcripts along with the matching mature miRNAs are given in Additional file 6.

A subset of 28 genes predicted as targets applying the holistic approach were immune response network genes (Table 3) that was not discovered using the SAV responsive set of genes as input in the target gene predictions. Although an association between these genes and SAV has not been reported, the *in silico* prediction analysis indicates that these genes may also be among the genes targeted by the DE miRNAs. The majority of these transcripts encode proteins with roles as activators (IRF1, IRF4, IRF5) and effectors (chemokine receptors) in the immune responses while a few of them are inhibitors (e.g. NFKappaB inhibitor) (Table 3).

**Table 3:** Predicted target genes with immune response related function from in silico analysis using the Atlantic salmon transcriptome as input (holistic apporach)

Gene	Gene function^1^	Predicted by four search tools^2^	Predicted by three search tools^3^	Reference^4^
Interferon regulatory factor 2-binding protein 2-A	Repressor, regulation of transcription, represses the NFAT1-dependent transactivation of NFAT-responsive promoters	miR-223_5p	−	NM_001139853.1
interferon regulatory factor 5 (IRF5)	Transcription factor activator that promote inflammation, innate immune system, Antiviral defense	miR-181c-5p	miR-462a-3p	NM_001139852.1
CD226 antigen precursor	Activator, Receptor involved in cytokine production, regulation of immune response in innate immune system	miR-146a-3p, miR-181c-5p	miR-7132-3p	NM_001139914.1
POU domain class 2-associating factor 1	Transcriptional coactivator, humoral immune response	miR-181c-5p	−	NM_001140075.1
Suppressor of IKK-epsilon	inhibitory role in virus- and TLR3-triggered IRF3, Inhibits TLR3-mediated activation of interferon-stim. response elements (ISRE)	miR-146a-3-3p, miR-146a-3p	miR-21a-2-3p, miR-21b-3p	NM_001140308.1
NF-kappa-B inhibitor epsilon	Inhibition of NFkB, innate immune system signaling pathway	miR-181c-5p	miR-146b-5p	NM_001140380.1
Interferon-induced protein 44 (IFI44)	GTP binding, antiviral defense	miR-21b-3p	miR-21a-2-3p	NM_001140400.1
C-C chemokine receptor type 9	Transcription coactivator binding, chemokine receptor activity, immune response	−	miR-181c-5p, miR-731-5p	NM_001140518.1
C-C motif chemokine 25 precursor	Effector, chemokine-mediated signaling pathway, immune response, inflammatory response	miR-155-5p	−	NM_001140865.1
C-X-C motif chemokine 10 precursor	Chemokine-mediated signaling pathway, effector, immune response, inflammatory response	miR-181c-5p	:miR-21b-3p	NM_001141028.1
Interleukin-20 receptor alpha chain precursor	Activator, cytokine-mediated signaling pathway, Interleukin 20 binding, immune system	−	miR-92a-3-5p	NM_001141080.1
C-C motif chemokine 21 precursor	Chemokine-mediated signaling pathway, effector, immune response, inflammatory response	miR-92a-3-5p	miR-21a-2-3p, miR-21b-3p	NM_001141267.2
interferon-induced, ds RNA-activated protein kinase	Activator and effector, Antiviral defense, Host-virus interaction, Immunity, Innate immunity, Transcription, Transcription regulation	miR-146b-5p	−	NM_001141332.2
C-C chemokine receptor type 3	Effector, chemokine receptor activity, inflammatory response, Host-virus interaction, cellular defense response	−	miR-7132-3p	NM_001173762.1
Interleukin-31 receptor A precursor	Transcription coactivator, activates STAT3, cytokine receptor activity, acute inflammatory response to antigenic stimulus	−	miR-1338-3p	NM_001173970.1
interferon regulatory factor 1 (IRF1) isoform 2	Activator, promote antiviral innate immunity response, Transcription regulation	miR-462b-5p	miR-7132-5p	NM_001252364.1
interleukin 1 receptor accessory protein precursor	Activator, IL-1 signaling pathway, Immunity, Inflammatory response, innate immune response	miR-2188-3p	−	NM_001123552.1
CCL4-like chemokine precursor	Effector, chemokine-mediated signaling pathway, immune response, inflammatory response	miR-146a-3-3p, miR-146a-3p	−	NM_001123618.1
C-C chemokine receptor type 6	C-C chemokine binding, cellular defense response, innate immune response	miR-181c-5p	−	NM_001139972.1
Interferon regulatory factor 4 (IRF4)	Transcriptional activator, type I interferon signaling pathway, binds to ISRE, immune system pathways	−	miR-731-5p	NM_001139982.1
Interleukin-13 receptor alpha-2 chain precursor	Cytokine receptor activity, immunoglobulin mediated immune response, negative regulation of immunoglobulin production	miR-731-3p	miR-731-5p	NM_001140169.1
C-X-C chemokine receptor type 3	Receptor for C-X-R chemokines, regulates biological processes such as inflammation, immunity, and would healing	miR-731-5p	miR-734-3p	NM_001140493.1
nuclear factor NF-kappa-B p100 subunit	Activator and repressor, transcr. factor activity, NF-kappaB signaling pathway, innate immune response, innflammatory response	−	miR-462a-3p	NM_001173583.1
toll-like leucine-rich repeat precursor	Activator, Immunity, Inflammatory response, Innate immunity	miR-731-5p	miR-2188-3p	NM_001123691.1
Macrophage migration inhibitory factor (MIF)	Inhibitor/surpressor, Inflammatory response, innate immune response, cell-mediated immunity	miR-155-5p	−	NM_001141547.1
BCL2/adenovirus E1B interacting protein 3-like	Effector, negative regulation of apoptotic process, Host-virus interaction, defense response to virus	miR-1338-3p, miR-146b-5p, miR-181c-5p, miR-731-5p	−	NM_001141679.1
TRAF2-binding protein	mediates the IRAK1 and TRAF6 interaction following IL-1 stimulation, I-kappaB kinase/NF-kappaB signaling	−	miR-462a-3p	NM_001173784.1
single Ig IL-1-related receptor precursor	Acute-phase response, neg. reg. of cytokine-mediated signaling pathway, neg. reg.of sequence-specific DNA binding transcription factor activity	miR-181c-5p, miR-462a-3p	−	NM_001173838.1

^1^Key words about gene function are based on gene function description in the http://www.uniprot.org/uniprot/ database. ^2^DE-miRs predicted to target the gene by all the four target search tools used (RNA-hybrid, TargetSpy, PITA, Miranda)

^3^DE-miRs predicted to target the gene by three of the four target search tools used. ^4^Genbank reference number

### Genome sequence analysis of DE miRNA genes to identify putative *cis*-elements

The genome sequence of the miRNA precursors and their location within the Atlantic salmon genome was described in Andreassen et al. [25], and an updated version of the Atlantic salmon genome sequence was recently uploaded in Genbank (Genbank # GCF_000233375.1). Together these sources were utilized to identify and analyze the upstream sequences of the DE miRNA genes. The 20 short mature DE miRNAs could be processed from 25 miRNA genes as some of the mature miRNAs could originate from highly similar duplicated miRNA genes. While the upstream genome sequence was not available for one of the genes (miRNA-155-2), the genome sequences from the other 24 miRNA genes could be included in the genome sequence analysis. They were all analyzed regarding presence of the consensus sequences of eight conserved promoter motifs known to bind immune-response-activated transcription factors (Additional file 7). The presence of such elements could indicate that their transcription was enhanced by transcription factors that are part of the innate immune response gene network.

The results from the search for response elements in the immediate upstream sequences of the DE miRNA genes are summarized in Table 4. Ten of the genes revealed TATA-box motifs, a finding in agreement with Long et al. [47]. More interestingly, two or more of the eight promoter motifs were observed in the immediate upstream sequences of all genes except miRNA-734 and miRNA-2188 that did not reveal any of the motifs in their upstream sequences. The miRNA-462a/731 gene cluster as well as the single miRNA-462b gene revealed the “classical” ISRE motif (ISRE-IRF1 in Table 4), the PU.1 motif and the GAS motif which have also been identified in the upstream sequence of the miRNA-462a/731 gene cluster in rainbow trout [28]. In addition, there were three more out of the eight promoter motifs present upstream of this gene cluster (Table 4). The “classical” ISRE motif was also present in the miRNA-146b and miRNA-155-1 genes along with three or more of the other motifs. In agreement with findings that miRNA-155 transcription is regulated through binding to NF-kappaB and ETS2 motifs [48], these motifs were also present in the Atlantic salmon miRNA-155-1 gene. In summary, the genome analysis showed that there was a high density of motifs known to bind transcription factors activated by the innate immune system in the upstream genome sequences of the DE miRNA genes. This indicates that transcription of many of the DE miRNA genes may be enhanced by some of the key transcription factors of the innate immune system. The two miRNA genes, miRNA-734 and miRNA-2188, that did not have any matches to the conserved motifs in their upstream sequences were the ones that revealed a decrease of their mature miRNAs following infection (Table 1).

**Table 4:** Transcription factor binding motifs in the upstream genome sequences of DE miRNA genes

miRNA gene	upstream TATA-box	ISRE-IRF1 M00972	IRF1 M00747	PU.1	GAS	IRF3 M00772	IRF8 M01665	NF-kappaB M00051	ETS2 M00340	Genbank Accesion #
miR462a/731 cluster	1	2	1	1	2	1	1	-	-	gi|925216714:63629639-63631038 (NC_027314.1)
ssa-mir-462b	1	2	1	1	1	2	1	-	-	gi|925216718:28307432-28308831 (NC_027312.1)
ssa-mir-146a-1	-	-	-	-	3	-	1	-	-	gi|925216706:4556929-4558189 (NC_027317.1)
ssa-mir-146a-2	-	-	-	-	2	-	-	-	-	gi|925216783:47301787-47303047 (NC_027300.1)
ssa-mir-146b	-	1	1	-	5	-	1	-	-	gi|925216704:2641623-2642883 (NC_027318.1)
ssa-mir-146a-3	-	-	3	-	3	-	-	-	-	gi|925216704:51691919-51693179 (NC_027318.1)
ssa-mir-21a-1	2	-	-	-	1	-	1	-	-	gi|925216727:116610294-116611574 (NC_027308.1)
ssa-mir-21a-2	1	-	-	-	1	-	2	-	-	gi|925216702:60966622-60967902 (NC_027319.1)
ssa-mir-21b-1	1	-	-	-	1	-	1	-	-	gi|925216769:50774080-50775360 (NC_027303.1)
ssa-mir-21b-2	-	-	1	-	1	1	1	-	-	gi|925216718:73813159-73814439 (NC_027312.1)
ssa-mir-1338	-	-	2	-	1	-	2	-	-	gi|925216772:45802741-45804012 (NC_027302.1)
ssa-mir-155-1	-	1	-	-	6	-	-	1	1	gi|925146043:6698-7958 (NW_012350889.1)
ssa-mir-92a-3	-	-	-	-	1	-	-	-	-	gi|925125935:50220-51479 (NW_012343521.1)
ssa-mir-7132b-1	2	-	-	1	4	-	-	-	-	gi|925216780:44866633-44867894 (NC_027301.1)
ssa-mir-7132b-2	3	-	-	1	1	-	-	-	-	gi|925216720:24179665-24180926 (NC_027311.1)
ssa-mir-7132a-1	-	-	1	1	1	-	1	-	-	gi|925216772:70457167-70458424 (NC_027302.1)
ssa-mir-7132a-2	-	-	1	1	1	-	-	-	-	gi|925216757:28942285-28943542 (NC_027305.1)
ssa-mir-734	-	-	-	-	-	-	-	-	-	gi|925216691:29709642-29710902 (NC_027324.1)
ssa-mir-181b	1	-	-	-	-	1	-	-	-	gi|925216772:43728947-43730207 (NC_027302.1)
ssa-mir-181c	1	-	-	-	2	-	-	-	-	gi|925216723:49524703-49525962 (NC_027310.1)
ssa-mir-2188-1	-	-	-	-	-	-	-	-	-	gi|925216702:26812353-26813612 (NC_027319.1)
ssa-mir-2188-2	-	-	-	-	-	-	-	-	-	gi|925216694:16936155-16937414 (NC_027323.1)
miR-223-1	1	-	1	-	4	-	1	-	-	gi|925216769:43698893-43700211 (NC_027303)
miR-223-2	-	-	1	-	2	1	1	-	-	gi|925216718:81008935-81010247 (NC_027312.1)

Consensus sequences to each of the nine cis-elements are given in Additional file 4

Genbank accession # gives the complete genome sequences spanning the miRNA genes analysed

## Discussion

### DE miRNAs may participate in negative feedback loops in the host innate immune system

The quantitative analysis of the results from the deep sequencing followed by RT-qPCR validations identified a subset of mature miRNAs that are differentially expressed following SAV infection in Atlantic salmon (DE miRNAs). The relative expression of the DE miRNAs was subsequently measured across three time points post onset of challenge in fish infected with either SAV2-i1, SAV3-i4 or SAV3-i6 (Figs. 3 and 4).

The DE miRNAs were predicted to target a number of key genes of the innate immune system. Some are among the important activators of the innate immune responses like the ATP-dependent RNA helicase DHX58 as well as the transcription factors IRF3 and IRF7. Other innate immune response network genes that were predicted as targets were interferon gamma (IFN-γ) and interferon stimulated genes like viperine (RSAD2), C–C motif chemokine 19, interferon-inducible protein Gig2-2-like, Gig2-7-like and myeloperoxidase (Table 2). These genes are all known to respond to SAV infection [38, 39]. Extending the search for putative target genes to the Atlantic salmon transcriptome identified 28 additional immune network genes as putative targets (Table 3). The majority of the putative target genes act as activators or effector molecules in the innate immune system pathway. If they are true target genes they are negatively regulated by DE miRNAs.

The role of the DE miRNAs in the host-virus interaction would not only depend on what target genes they regulate, but would also depend on what time post viral infection their expression changes as well as the direction of change. Several recent reviews [11, 49–51] suggest that some miRNAs targeting activator and effector transcripts could have a role in promoting early immune response if their expression was decreased at the early stage of an immune response. In the late phase of inflammation, some miRNAs that target activator and effector transcripts have been suggested to have another role. In this phase increased expression of these miRNAs may help prevent pathological inflammatory processes. In this role the miRNAs stabilize and prevent further increase of activator and effector transcripts, and both the targets and miRNAs would have increased expression compared to the normal cellular state. The expression of some miRNAs believed to negatively regulate the immune response in the late phase of infection seem to be induced by immune network transcription factors. In such cases the miRNAs participate in negative feedback loops as their expression is enhanced by their targets [11, 49].

The viral loads increased across the three first time points (week 1, 2 and 3 poc) in all the SAV groups, but seemed to decline at week 4 poc in SAV3-i6. The viral loads in the two other groups also seemed to have reached their maximum level at week 4 poc as the increases were much less pronounced from week 3 to week 4 poc (Fig. 1). These observations are in agreement with findings in Taksdal et al. [32] where the viral loads declined in all SAV groups after week 4 poc. Ten of the miRNAs (e.g. the eight miRNAs in Fig. 3) showed minor increases across the first time point (week 2 poc), but larger increases towards the last time point (week 4 poc). The findings from measurements of viral load showed that all fish had been infected for more than a week at the last time point (week 4 poc, Fig. 1). Comparing the increases in viral loads with the change in miRNA expression the latter appear as a time delayed response to viral infection. This could indicate that the purpose of the increases, resulting in a high expression of the DE miRNAs more than a week post maximum measurements of viral loads, were to negatively regulate the expression of inflammatory mediators to prevent excess inflammation [50, 51]. Many DE miRNAs showed a time delayed increase of the DE miRNAs (Fig. 3) and they were predicted to target immune response activators. This could suggest that their role were to prevent a prolonged or elevated immune response that would be harmful to the host [11, 49].

The initial screening to identify DE miRNAs was carried out in materials from the latest time point post challenge (week 4 poc). It would therefore be expected that the majority of the miRNAs identified would be those involved in preventing pathological inflammation and tissue damage. Since we did not include earlier time points in the initial screening for DE miRNAs, we cannot rule out that other miRNAs are important regulators early in the infection. The role of such early responding miRNAs could be to promote an immune response. The decreased expression at the early time points of a few DE miRNAs identified in this study, e.g. the mature miRNAs from the miRNA 21 family (Fig. 4), could indicate that they may have such a role where they contribute to promote the early immune response. The downregulation at the early time points in the SAV2-i1 group of the mature miRNAs from the miRNA 21 family (Fig. 4) would be expected to contribute to an immediate increase in the immune response as downregulation of the miRNAs would lead to a proportional downregulation of the translational repression of the predicted target gene IRF3 (Table 2). The downregulation of another miRNA, ssa-miR-734-3p, was detected in the DESeq2 analysis, but could not be validated by RT-qPCR in our study (Table 1). However, if this miRNA is downregulated in the very early phase of infection this would also lead to an increased immune response as it was predicted to target viperine and interferon gamma (Table 2).

Analysis of the genomic sequences spanning the DE miRNA genes revealed a high density of response elements that may bind transcription factors associated with an innate immune response. Similar findings have been reported by Schyt et al. [28] for the teleost specific miRNA 462/731-gene cluster analysed in rainbow trout. Other conserved miRNA genes, like miRNA-146, miRNA-181 and miRNA-155 are also known to be INF-regulated in vertebrates with increased expression upon stimulation [11]. If the expression of the DE miRNAs identified in our study is enhanced by binding transcription factors activated by the immune response, this means that many of the DE miRNAs are the effector molecules participating in negative feedback loops. A recent study of miR-731-5p in Japanese flounder [44] demonstrated such a negative feedback where miR-731-5p was shown to target the transcription factor IRF7. The analyses of target genes in our study showed that, not only was ssa-miR-731-5p predicted to target the salmon ortholog of IRF7, but it was also predicted to target the ATP-dependent RNA helicase DHX58 (RIG-I-like 3). The RIG-I-like receptors are believed to be upstream activators of IRF7 [39]. Targeting not only IRF7, but also the upstream activator of IRF7, an increased expression of ssa-miR-731-5p would, as described in Zhang et al. [44], negatively regulate the immune response.

Together, the target gene predictions and the analysis of upstream genome sequences of the miRNA genes suggest that many of the DE miRNAs are important participants of the immune system gene pathways and some may act in negative feedback loops to maintain immune homeostasis. However, as demonstrated in our study, the expression of these DE miRNAs may differ depending on what kind of virus subtype that causes the immune response. Even virus infection caused by virus isolates belonging to the same subtype (SAV3-i4 and SAV3-i6) resulted in significant differences in expression of most of the DE miRNAs.

### Different expression patterns of the DE miRNAs may help explain differences in mortality

The high expression at the late time point and the predicted target genes suggest that the ten miRNA (eight of these shown in Fig. 3) may act to prevent excess inflammation. The expression of these miRNAs (ssa-miR-462a-5p and 3p, ssa-miR-731-5p and 3p, ssa-miR-146a-3p, ssa-miR-146b-5p, ssa-miR223-5p, ssa-miR-155-5p, ssa-miR-181c-5p and ssa-miR7132-3p) were higher in the fish infected with SAV3-i6 (Fig. 3, Additional file 3) than in the other SAV3 isolate (SAV3-i4) at week 4 poc. This observation is interesting as Taksdal et al. [32] reported that there was a difference in mortality between the two isolates of SAV3 with a higher mortality in the SAV3-i4 group than in the SAV3-i6 group that could not be explained by their results. A higher expression of the ten DE miRNAs in the SAV3-i6 group than in the SAV3-i4 group could lead to a more pronounced down regulation of the inflammatory process in the SAV3-i6 group. The effect of this could be less pathological inflammation in the cardiac tissue in the following weeks that might result in a reduced mortality in the SAV3-i6 group compared to the SAV3-i4 group. The observation that ssa-miR-2188-3p has a significantly lower expression in the SAV3-i4 group than in the SAV3-i6 group (Fig. 4) could also contribute to a higher mortality in the SAV3-i4 group. The two predicted targets of this miRNA promote inflammatory response and cell death (Table 2). Thus, a prolonged high expression of these predicted target genes would not benefit the host. The observed differences in expression of DE miRNAs between the SAV3-i4 and SAV3-i6 group are therefore in agreement with, and offer an explanation to, the observed differences in mortality between fish infected with these isolates of SAV3 subtype.

Fish challenged with SAV2-i1 was reported with a significantly lower mortality than those challenged with the two SAV3 isolates [32]. The SAV2-i1 group did, like the SAV3-i6 group, show a significantly higher expression at the latest time point in some miRNAs compared to the SAV3-i4 group (ssa-miR-462a-5p, ssa-miR155-5p and ssa-miR-7132-3p, Fig. 3). However, in other miRNAs they revealed similar expression levels as those from the SAV3-i4 group, and comparing the SAV2-i1 group to the SAV3-i6 group all ten miRNAs (Fig. 3) showed a much lower expression in the SAV2-i1 group. The miRNA expression at the late time point in infection (week 4 poc) in these miRNAs could therefore not explain a mortality rate that was lower in the group infected by SAV2-i1 than in both of the groups infected with the SAV3 subtypes (SAV3-i6 and SAV3-i4). However, the expression of other miRNAs at the early time points may offer an explanation to these differences in mortality rate. There was a decreased expression in the miRNAs from the miRNA 21 family at week 2 and 3 poc in the SAV2-i1 group (Fig. 4). In contrast, there was an increase in expression across the same time points in the groups challenged with both isolates from the SAV3 subtype. These differences in expression, a reduction of miRNAs from the miRNA 21 family in the SAV2-i1 group *versus* an increase of these miRNAs in both of the SAV3 groups would lead to a faster and much more pronounced downregulation of the negative repression of the target gene in the SAV2-i1 group. The predicted target gene is IRF3, a constitutively expressed key activator of the innate immune system [52]. Thus, the differences in miRNA 21 family expressions could contribute to a faster and more effective early immune response in fish infected by SAV2-i1 and lead to a lower mortality in this group.

Although the observed differences in expression between DE miRNAs may offer some explanations to differences in virulence and mortality observed in fish infected by different SAV isolates they rely on the correctness of the target gene predictions. Thus, an obvious way forward would be to validate the target genes of the identified DE miRNAs and further explore the miRNA-target gene interactions by experimental approaches.

## Conclusion

This is the first miRNA profiling study in Atlantic salmon that has identified and characterized cellular miRNAs with modulated expression following virus infection indicating that they are important participants in the virus-host interaction. The target gene predictions and analysis of upstream genome sequences suggests that many of the miRNAs may regulate innate immune responses as effector molecules in negative feedback loops. Our findings represent a first step in exploring and suggesting a role for Atlantic salmon miRNAs in virus-host interactions. Further functional studies will help to disclose the particular role of these miRNA genes, and may contribute more knowledge on how these miRNAs act in concert with other regulator molecules to fine-tune the innate immune system responses.

## Materials and Methods

### Materials

A challenge trial was carried out where healthy Atlantic salmon (smolt) were exposed to SAV by cohabitating with salmon shedders injected with virus (carrier fish). The cohabitant challenge experiment and sampling of tissue is described in detail in Taksdal et al. [32]. In short, the fish used for the challenge experiment was unvaccinated, clinically healthy Atlantic salmon from VESO Vikane hatchery. Health conditions was also confirmed by examining ten fish for common fish viruses. They all tested negative for piscine myocarditis virus (PMCV), piscine reovirus (PRV), infectious pancreatic necrosis virus (IPNV) and SAV. The fish was challenged with 3 SAV-isolates derived from independent outbreaks of Pancreas Disease in Norwegian fish farms, either marine SAV2 (SAV2-i1) or one of two isolates of SAV3; SAV3-i4 and SAV3-i6 [32].

Cardiac tissue samples were collected from the cohabitating (non-injected) fish. Co-habitant samples were collected at four time points; at the initiation of the experiment (controls), at week one, two, three and four post onset of the challenge experiment. Eight fish collected at the initiation of the experiment were used as healthy controls, while nine fish, three from each SAV group was collected at week 1 poc. Nine fish challenged with either SAV2-i1, SAV3-i4 or SAV3-i6 were sampled at week two, three and four poc. Together, cardiac tissue from 98 fish was sampled. All samples from controls, week one, two, three and four poc were analysed to measure viral load by RT-qPCR. All samples from week 1 poc, four samples from fish challenged with SAV2-i1 and one sample from fish challenged with SAV3-i4 at week 2 poc were SAV negative and were removed from further analysis of miRNA expression. The samples from the remaining individuals at week 2 poc, and all fish from week 3 and 4 poc, tested positive for SAV (*n* = 78). The eight control samples and the SAV positive samples from other time points (total of 84 samples) were all included in our study of miRNA expression (RT-qPCR of miRNA).

Three healthy controls and three samples collected at week 4 poc from the SAV3-i4 group were used for deep sequencing and DESeq2 analysis. Fish infected with SAV3-i4 were choosen as this was the SAV isolate associated with the highest mortality rate [32]. The late time point (week 4 poc) was choosen as the virus loads were high and 100% of fish from all three groups were infected at this time point. The experimental study was approved by the National Research Authority in Norway (NARA). All salmon used for sampling in the experiment were euthanized according to standard protocols approved by the Norwegian Food Safety Authorities prior to sampling.

## Methods

### Small RNA isolation

Sampling of cardiac tissue was carried out as described in Taksdal et al. [32]. Samples were fixed in RNA later (Life technologies, Carlsbad, USA) immediately after sampling. Total RNA was isolated from each of the samples by use of the mirVana miRNA isolation kit (Ambion, Life Technologies, Carlsbad, CA, USA) following the manufacturer’s protocol. The RNA concentration and purity was determined using Nanodrop spectrophotometer (Thermo Fisher Scientific, WI, USA) following the manufacturer’s protocol. The concentration of total RNA in samples ranged from 21–255 ng/μl (total volume 100 μl) and with A260/280 ratios above 1,9. The integrity of the RNA in samples used for deep sequencing was analyzed using the Agilent Bioanalyzer system (Agilent, CA, USA). The RIN values were from 6.8 to 10 (Additional file 1).

### RT-qPCR detection of SAV

SAV in the cardiac samples was detected by RT-qPCR with a Qiagen Onestep RT-PCR kit using TaqMan assay with primers and probe targeting the nsP1 gene as described in Hodeland and Endresen [53]. Viral load (Ct values) was measured by RT-qPCR in all samples from controls, week one, two, three and four poc (*n* = 98).

### Library construction, deep sequencing and quantitative analysis

The library construction was performed at the Norwegian Genomics Consortium’s genomics core facility. The Illumina TruSeq Small RNA Library Preparation Kit (Illumina, San Diego, USA) was used in the preparation of the libraries as described by the manufacturer with 1 ug total RNA input. After adapter ligation and cDNA synthesis the products were purified on a gel and the fractions between 145 and 160 bp were used for sequencing. The small RNA libraries constructed from six samples (three from controls and three from the SAV3-i4 group sampled at week 4 post onset of challenge experiment) were successfully subjected to next generation sequencing using Illumina Genome Analyzer IIx sequencing platform as described in Andreassen et al. [25]. Reads quality were assessed using FastQC toolkit (http://www.bioinformatics.babraham.ac.uk/projects/fastqc). Cutadapt [54] was used for trimming of adapter sequences from raw sequence reads and removal of adapter-only sequences (5’TGGAATTCTCGGGTGCCAAGGAACTCCAGTCAC 3’). Finally, an additional filtering of reads outside of 18–25 nucleotide range was applied on all samples. All deep sequencing samples have been submitted to the NCBI Sequence Read Archive database (SRA) and accession numbers are given in Additional file 1.

Next, the reads from each of the six samples were aligned to a set of miRNAs that consisted of all known *Salmo salar* mature miRNAs [25, 26] using Novoalign (http://www.novocraft.com/products/novoalign/). Custom made scripts were used to report number of successfully assigned reads to each miRNA. The reads mapping with edit distance 1 or less to the mature reference sequences were considered true mature miRNAs. Differentially expressed miRNAs were identified with DESeq2 package [33]. Rows with less than 2 reads for each condition were discarded from the analysis. A threshold of p-adjusted value <0.1 (adjusted according to Benjamini-Hochberg procedure) was applied to report differentially expressed miRNAs. The two groups compared were controls (three samples) and SAV3-i4 infected individuals (three samples) from week 4 poc.

### cDNA synthesis and RT-qPCR of miRNAs

The miScript assays were used for cDNA synthesis (miScript II RT Kit) and qPCR (miScript SYBR Green PCR kit) as described by the manufacturer (Qiagen, Hilden, Germany). The reverse primer (universal primer) was provided with the miScript qPCR kit while the forward primer was custom designed and specific to each of the mature miRNA gene assays. The sequence of the forward primers were designed based on the mature sequence of each of the miRNA genes to be amplified [25]. All forward primers were purchased from Sigma Aldridge. They were purified by desalt only and provided as liquid solution of 100 μM. The primers were diluted to 10 μM for use in each of the qPCR assays. All forward primer sequences are given in Additional file 2. The qPCR analysis was run on an Mx3000p (Stratagene, Agilent Technologies, USA). The qPCR reaction mixture consisted of 12.5 μL 2x Quantitec Syber Green Master Mix, 2.5 μL 10x miScript Universal Primer, 2.5 μL of 10 μM forward miRNA gene specific primer, 5 μL Rnase free water, and 2.5 μL cDNA (template). Amplification was performed in 96-well plates. The following program was used: one thermal cycle at 95 °C for 15 min followed by 40 cycles of 94 °C for 15 sec, 55 °C for 30 sec and 70 °C for 30 sec. The cybergreen assay module was used for qPCR analysis. This module includes a final melting point analysis that follows the 40 cycles of quantitative PCR. A manual inspection of the plots from the melting point analysis of all miRNA gene assays tested was carried out to verify that forward primers were specific (data not shown).

In all cases of measurements of miRNA expression by RT-qPCR, the samples that were compared were run in the same set-up (within run measurements). Three miRNAs ssa-miR-25-3p, ssa-miR-455-5p and ssa-miR-17-5p were used as reference genes [27]. Results (Ct-values) of target miRNAs were normalized by use of the geometric mean from the measurements of the three reference genes [55]. The relative increase in expression in target tissue of each miRNA was calculated using the principle of the ΔΔCt-method [35]. Applying this method the average relative difference in target tissue was calculated as the difference between mean normalized Ct from target tissue and mean normalized Ct from reference tissue. To test whether the relative differences in expression revealed by the ΔΔCt-method between controls and SAV infected groups was significant we performed Mann-Whitney tests using the statistical package SPSS. Likewise, Mann-Whitney tests were also used to test for significant difference in expression of miRNAs at the same time point between the three SAV groups. P-values were adjusted by use of the Benjamini-Hochberg procedure taking into account there were 153 comparisons between SAV groups at different time points (P-adjusted values equal to a 0.05 significance level). Test for correlation between estimates of relative differences by DESeq2 and by RT-qPCR of the 17 mature miRNAs analysed by both methods was carried out by use of Spearman’s nonparametric test for correlation (SPSS package).

### miRNA target gene predictions

The mature sequences from the DE miRNAs were used as input in the target gene prediction analysis. The 3’UTRs from two sets of genes were used as input for target genes. One set was genes reported as responding to SAV infection and associated with immune response or response to viral infection [38, 39]. In addition, the Atlantic salmon orthologs to some genes that are key regulators of immune responses and reported in other species as targets of the same miRNAs as those identified in our study were included in this set of genes [40–45]. Applying these selection criteria based on prior knowledge about their relevance to SAV infection and immune response (knowledge based approach) a total of 50 genes were analysed to detect target site matches (Additional file 4). The other set of genes used as input were 3’UTRs from all Atlantic salmon mRNA transcripts in the Refseq database in Genbank (https://www.ncbi.nlm.nih.gov/). This holistic approach could possibly identify target genes among immune network genes not previously known to respond to SAV infection.

The *in silico* analysis to identify target genes were carried out by use of four different target gene prediction tools; RNAhybrid, TargetSpy, PITA and Miranda. RNAhybrid analysis was performed with conditions helix constraint 2–8 and no G:U in seed allowing only target genes that had perfect “seed” matches to be detected [36]. Minimum free energy threshold for RNA hybrids was set to -18 kcal/mol to retrieve results (target site matches) from RNA hybrids that had a high stability [56]. RNA hybrid was used to generate the figure in Additional file 5. The target site analysis with the tools TargetSpy, PITA and Miranda was carried out applying default settings using the sRNAtoolbox (http://bioinfo5.ugr.es/srnatoolbox/mirconstarget/)[37].

### Functional motif analysis of the genome sequences spanning the DE miRNA genes

The upstream genome sequences of all DE miRNA genes were retrieved by using precursor sequences [25] as input in BLASTn searches against the current version of the *Salmo salar* genome sequence in Genbank (Genbank # GCF_000233375.1). Several of the mature miRNAs could originate from duplicated miRNA genes. Thus, the total number of miRNA genes analysed was 24. One thousand base-pairs of the upstream sequence and 200 base-pairs of the downstream sequence from the mature 5p sequences of each the DE miRNA genes were analysed regarding presence of conserved response elements known to bind key transcription factors from the innate immune system gene network. Consensus sequences of vertebrate response elements were retrieved from the MotifMap database (http://motifmap.ics.uci.edu/). In addition, Salmonid specific motifs were retrieved from Robertsen [57] and Schyth et al. [28]. The consensus sequences of the nine response elements used for upstream genome analysis are given in Additional file 7. The consensus sequences of conserved immune related response elements (ISRE-IRF, NF-kappaB and ETS2), the TATA-box consensus sequence as well as the response elements GAS and PU1 characterized in a study of the rainbow miRNA 462/731 gene cluster [28] are all detected with the general consensus sequences used in our genome sequence analysis. ISRE and NFkappaB motifs in zebrafish are also detected using these motifs [58, 59] indicating that the consensus sequence applied detect these motifs in teleost fish (including Atlantic salmon). Presence of any motif was detected by use of Sequencher software (Gene Codes Corporation, Michigan, USA).

## Additional files

Additional file 1: shows descriptive data from deep sequencing of six samples including number of size filtered and adapter trimmed reads, and reads mapped as ssa-miRNAs in each sample. (XLSX 17 kb)

Additional file 2: shows all primer sequences of primers used in the RT-qPCR along with R-square values and efficiency measurements. (XLSX 12 kb)

Additional file 3: gives a complete overview of all results from RT-qPCR analysis showing the magnitude of change in each SAV-group compared to controls at each of the time points along with measurements of significance (adjusted p-values). All results from comparing different SAV groups within same time points are also summarized in Additional file 2. (XLSX 20 kb)

Additional file 4: shows all SAV responsive immune network genes that were included in the target gene analysis against the DE miRNAs (knowledge based approach. (XLSX 15 kb)

Additional file 5: shows the predicted RNA hybrid formed between ssa-miR-21b-3p and the nucleotides 805–827 in the IRF3 transcript’s 3’UTR. (DOCX 16 kb)

Additional file 6: shows complete results from target gene predictions using all Atlantic salmon transcripts in Refseq, Genbank as input in the *in silico* analysis (holistic approach). (XLSX 220 kb)

Additional file 7: gives the consensus sequences of all promotor response elements included in the analysis of upstream miRNA gene sequences. (XLSX 11 kb)

## Abbreviations

DE miRNAs: differentially expressed miRNAs
miRNA: microRNA
poc: post onset of challenge experiment
SAV2-i1: salmonid alphavirus subtype 2 isolate 1
SAV3-i4: salmonid alphavirus subtype 3 isolate 4
SAV3-i6: salmonid alphavirus subtype 3 isolate 6
