# Supplementary material for: Identification of differentially expressed Atlantic salmon miRNAs responding to salmonid alphavirus (SAV) infection
Source: BMC Genomics. 2017 May 4;18:349. doi: 10.1186/s12864-017-3741-3 (PMC5418855; doi:10.1186/s12864-017-3741-3)
Supplement: Supplementary file 5 — shows the predicted RNA hybrid formed between ssa-miR-21b-3p and the nucleotides 805–827 in the IRF3 transcript’s 3’UTR. (DOCX 16 kb) [file 12864_2017_3741_MOESM5_ESM.docx]

**Additional file 5.**


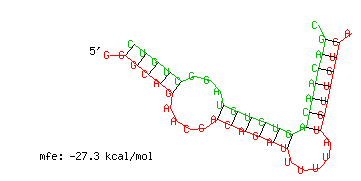


The predicted RNA hybrid formed between ssa-miR-21b-3p and the nucleotides 805-827 in the IRF3 transcript’s 3’UTR. The minimum free energy is -27.3 kcal/mol.
